# Supplementary material for: Host-parasite coevolution promotes innovation through deformations in fitness landscapes
Source: eLife. 2022 Jul 6;11:e76162. doi: 10.7554/eLife.76162 (PMC9259030; doi:10.7554/eLife.76162)
Supplement: Supplementary file 1. [file elife-76162-supp1.docx]

**Supplementary File for**

**Host-parasite coevolution promotes innovation through deformations in fitness landscapes**

Animesh Gupta, Luis Zaman, Hannah M. Strobel, Jenna Gallie, Alita R. Burmeister, Benjamin Kerr, Einat Tamar, Roy Kishony*, Justin R. Meyer*

*Corresponding authors: Roy Kishony and Justin R. Meyer

Email: rkishony@tx.technion.ac.il and jrmeyer@ucsd.edu

**This file contains all the supplementary tables.**

**Supplementary file 1a.** Reconstruction of the list of mutations in 24 phage isolates that evolved to target the OmpF receptor in the large-scale coevolution experiment by Meyer *et al.* (1). Highlighted mutations were chosen to form the genotype space for the 10-dimensional fitness landscape in Fig. 1. The first five and last five mutations fall within two 100-nucleotide windows for Mi-Seq 100 base paired-end sequencing.

**Mutations**

**in *J***

| **λgenotypes which evolved OmpF+ function in (*1*)** | T487C | C494T | C599T | A1747G | C2879T | A2966G | C2969T | A2975C | C2988A | A2989G | T2991G | C2999T | C3033T | A3034G | C3119T | T3143A | C3147G | A3158A | C3227T | T3230C | A3233G | T3248C | C3310T | G3319A | A3320G | T3321G | T3321A | A3364T | T3380C |
| --- | --- | --- | --- | --- | --- | --- | --- | --- | --- | --- | --- | --- | --- | --- | --- | --- | --- | --- | --- | --- | --- | --- | --- | --- | --- | --- | --- | --- | --- |
| A7 |  |  |  |  |  |  |  |  |  |  |  |  |  |  |  |  |  |  |  |  |  |  |  |  |  |  |  |  |  |
| A8 |  |  |  |  |  |  |  |  |  |  |  |  |  |  |  |  |  |  |  |  |  |  |  |  |  |  |  |  |  |
| A12 |  |  |  |  |  |  |  |  |  |  |  |  |  |  |  |  |  |  |  |  |  |  |  |  |  |  |  |  |  |
| B2 |  |  |  |  |  |  |  |  |  |  |  |  |  |  |  |  |  |  |  |  |  |  |  |  |  |  |  |  |  |
| C2 |  |  |  |  |  |  |  |  |  |  |  |  |  |  |  |  |  |  |  |  |  |  |  |  |  |  |  |  |  |
| C3 |  |  |  |  |  |  |  |  |  |  |  |  |  |  |  |  |  |  |  |  |  |  |  |  |  |  |  |  |  |
| D3 |  |  |  |  |  |  |  |  |  |  |  |  |  |  |  |  |  |  |  |  |  |  |  |  |  |  |  |  |  |
| D4 |  |  |  |  |  |  |  |  |  |  |  |  |  |  |  |  |  |  |  |  |  |  |  |  |  |  |  |  |  |
| D6 |  |  |  |  |  |  |  |  |  |  |  |  |  |  |  |  |  |  |  |  |  |  |  |  |  |  |  |  |  |
| D7 |  |  |  |  |  |  |  |  |  |  |  |  |  |  |  |  |  |  |  |  |  |  |  |  |  |  |  |  |  |
| D9 |  |  |  |  |  |  |  |  |  |  |  |  |  |  |  |  |  |  |  |  |  |  |  |  |  |  |  |  |  |
| E3 |  |  |  |  |  |  |  |  |  |  |  |  |  |  |  |  |  |  |  |  |  |  |  |  |  |  |  |  |  |
| E4 |  |  |  |  |  |  |  |  |  |  |  |  |  |  |  |  |  |  |  |  |  |  |  |  |  |  |  |  |  |
| E11 |  |  |  |  |  |  |  |  |  |  |  |  |  |  |  |  |  |  |  |  |  |  |  |  |  |  |  |  |  |
| E12 |  |  |  |  |  |  |  |  |  |  |  |  |  |  |  |  |  |  |  |  |  |  |  |  |  |  |  |  |  |
| F5 |  |  |  |  |  |  |  |  |  |  |  |  |  |  |  |  |  |  |  |  |  |  |  |  |  |  |  |  |  |
| F7 |  |  |  |  |  |  |  |  |  |  |  |  |  |  |  |  |  |  |  |  |  |  |  |  |  |  |  |  |  |
| F8 |  |  |  |  |  |  |  |  |  |  |  |  |  |  |  |  |  |  |  |  |  |  |  |  |  |  |  |  |  |
| G4 |  |  |  |  |  |  |  |  |  |  |  |  |  |  |  |  |  |  |  |  |  |  |  |  |  |  |  |  |  |
| G9 |  |  |  |  |  |  |  |  |  |  |  |  |  |  |  |  |  |  |  |  |  |  |  |  |  |  |  |  |  |
| H5 |  |  |  |  |  |  |  |  |  |  |  |  |  |  |  |  |  |  |  |  |  |  |  |  |  |  |  |  |  |
| H8 |  |  |  |  |  |  |  |  |  |  |  |  |  |  |  |  |  |  |  |  |  |  |  |  |  |  |  |  |  |
| H9 |  |  |  |  |  |  |  |  |  |  |  |  |  |  |  |  |  |  |  |  |  |  |  |  |  |  |  |  |  |
| H12 |  |  |  |  |  |  |  |  |  |  |  |  |  |  |  |  |  |  |  |  |  |  |  |  |  |  |  |  |  |

**Supplementary file 1b.** Result of Tukey’s significant test comparing for difference of means between different types of simulations in Fig 1c. The simulation treatments that were significantly different from each other are marked in red.

| **Difference of levels** | **Adjusted P-value** | **Difference of levels** | **Adjusted P-value** | **Difference of levels** | **Adjusted P-value** |
| --- | --- | --- | --- | --- | --- |
| *malT^—^* - 0.1 | 0.0124 | 0.1 - 0.8 | 0.9907 | 0.4 - 0.5 | 0.9838 |
| *malT^—^* - 0.2 | 0.2657 | 0.1 - 0.9 | 0.9585 | 0.4 - 0.6 | 0.1295 |
| *malT^—^* - 0.3 | 0.0003 | 0.2 - 0.3 | 0.4122 | 0.4 - 0.7 | 0.3106 |
| *malT^—^* - 0.4 | 0.0691 | 0.2 - 0.4 | 0.9999 | 0.4 - 0.8 | 0.8427 |
| *malT^—^* - 0.5 | 0.0023 | 0.2 - 0.5 | 0.7988 | 0.4 - 0.9 | 0.6969 |
| *malT^—^* - 0.6 | <0.0001 | 0.2 - 0.6 | 0.0269 | 0.5 - 0.6 | 0.7499 |
| *malT^—^* - 0.7 | <0.0001 | 0.2 - 0.7 | 0.0859 | 0.5 - 0.7 | 0.9384 |
| *malT^—^* - 0.8 | 0.0004 | 0.2 - 0.8 | 0.4676 | 0.5 - 0.8 | 1.0000 |
| *malT^—^* - 0.9 | 0.0002 | 0.2 - 0.9 | 0.3106 | 0.5 - 0.9 | 0.9989 |
| 0.1 - 0.2 | 0.9733 | 0.3 - 0.4 | 0.7988 | 0.6 - 0.7 | 1.0000 |
| 0.1 - 0.3 | 0.9838 | 0.3 - 0.5 | 0.9999 | 0.6 - 0.8 | 0.9585 |
| 0.1 - 0.4 | 0.9999 | 0.3 - 0.6 | 0.9733 | 0.6 - 0.9 | 0.9907 |
| 0.1 - 0.5 | 1.0000 | 0.3 - 0.7 | 0.9989 | 0.7 - 0.8 | 0.9976 |
| 0.1 - 0.6 | 0.4122 | 0.3 - 0.8 | 1.0000 | 0.7 - 0.9 | 0.9999 |
| 0.1 - 0.7 | 0.6969 | 0.3 - 0.9 | 1.0000 | 0.8 – 0.9 | 1.0000 |

**Supplementary file 1c.** Mutations and their corresponding labels in λ genotypes isolated from population D7 in Meyer *et al.* (1). Red asterisks indicate the particular mutation in a genotype’s description in Fig. 3a and Supplementary Table 4.

| **Mutations**  **λ Isolates** | T2991G  (A) | A3031G  (A*) | A3034G  (E) | G3319A  (B) | A3320G  (A**) | T3321A  (D) | T3380C  (C) |
| --- | --- | --- | --- | --- | --- | --- | --- |
| WT |  |  |  |  |  |  |  |
| A |  |  |  |  |  |  |  |
| A* |  |  |  |  |  |  |  |
| A** |  |  |  |  |  |  |  |
| AB |  |  |  |  |  |  |  |
| ABC |  |  |  |  |  |  |  |
| ABCDE |  |  |  |  |  |  |  |

**Supplementary file 1d.** Relative abundance of λ genotypes present at different times in population D7. This data was used to create the Muller plot in Fig. 3a.

| Day | Total number of plaques picked | Genotype | Frequency of the genotype |
| --- | --- | --- | --- |
| 0 | - | WT | 1 |
| 1 | 1 | A | 1 |
| 2 | 1 | A | 1 |
| 3 | 1 | A | 1 |
| 4 | 5 | A | 1 |
| 5 | 5 | A | 1 |
| 6 | 11 | A | 0.273 |
|  |  | A** | 0.636 |
|  |  | A* | 0.091 |
| 7 | 11 | A** | 0.818 |
|  |  | ABC | 0.091 |
|  |  | A**C | 0.091 |
| 8 | 11 | ABC | 0.273 |
|  |  | A**C | 0.727 |
| 9 | 5 | ABC | 0.2 |
|  |  | A**C | 0.8 |
| 10 | 5 | ABC | 1 |
| 11 | 5 | A**C | 0.2 |
|  |  | ABC | 0.2 |
|  |  | ABCDE | 0.6 |
| 12 | 5 | ABCDE | 1 |

**Supplementary file 1e.** Mutations present in λ isolates from day 26 of the coevolutionary replay experiment initiated with a) ancestor host and b) *malT^—^* host (corresponding to Fig. 4a and 4b). Two strains (*a* and *b*) were isolated from each population and the active region of *J* (roughly between nucleotide position 2,600 and the end) sequenced. Replicates marked in red indicate populations that evolved OmpF-function. Canonical mutations for evolution of OmpF function are bolded. λ evolved more of both the total number of mutations and total number of canonical mutations in replicate populations initiated with ancestor host than with the evolved *malT^—^* host (statistics for difference in total number of mutations: $t=5.37, d.f. =11, P=0.0002$, statistics for difference in total number of canonical mutations: $t=3.18, d.f. =11, P=0.0088$; both were tested using two-sample t-test with unequal variances assumed).

**Mutations**

**in *J***

|  | A2866T | T2908A | G2921A | G2966T | C2969T | C2988A | C2988G | A2989G | T2991G | T2993C | C2999T | A3031G | **A3034G** | C3119T | C3147G | G3226T | C3227T | T3230C | C3310T | **G3319A** | **T3321G** | **T3321A** | T3331C | **Total number of mutations** |
| --- | --- | --- | --- | --- | --- | --- | --- | --- | --- | --- | --- | --- | --- | --- | --- | --- | --- | --- | --- | --- | --- | --- | --- | --- |
| 3a |  |  |  |  |  |  |  |  |  |  |  |  |  |  |  |  |  |  |  |  |  |  |  | 3 |
| 3b |  |  |  |  |  |  |  |  |  |  |  |  |  |  |  |  |  |  |  |  |  |  |  | 3 |
| 4a |  |  |  |  |  |  |  |  |  |  |  |  |  |  |  |  |  |  |  |  |  |  |  | 8 |
| 4b |  |  |  |  |  |  |  |  |  |  |  |  |  |  |  |  |  |  |  |  |  |  |  | 8 |
| 5a |  |  |  |  |  |  |  |  |  |  |  |  |  |  |  |  |  |  |  |  |  |  |  | 4 |
| 5b |  |  |  |  |  |  |  |  |  |  |  |  |  |  |  |  |  |  |  |  |  |  |  | 4 |
| 6a  **ancestor host** |  |  |  |  |  |  |  |  |  |  |  |  |  |  |  |  |  |  |  |  |  |  |  | 5 |
| 6b |  |  |  |  |  |  |  |  |  |  |  |  |  |  |  |  |  |  |  |  |  |  |  | 5 |
| 8a |  |  |  |  |  |  |  |  |  |  |  |  |  |  |  |  |  |  |  |  |  |  |  | 4 |
| 8b |  |  |  |  |  |  |  |  |  |  |  |  |  |  |  |  |  |  |  |  |  |  |  | 3 |
| 9a |  |  |  |  |  |  |  |  |  |  |  |  |  |  |  |  |  |  |  |  |  |  |  | 4 |
| 9b |  |  |  |  |  |  |  |  |  |  |  |  |  |  |  |  |  |  |  |  |  |  |  | 4 |
| 10a |  |  |  |  |  |  |  |  |  |  |  |  |  |  |  |  |  |  |  |  |  |  |  | 4 |
| 10b |  |  |  |  |  |  |  |  |  |  |  |  |  |  |  |  |  |  |  |  |  |  |  | 4 |
| 11a |  |  |  |  |  |  |  |  |  |  |  |  |  |  |  |  |  |  |  |  |  |  |  | 6 |
| 11b |  |  |  |  |  |  |  |  |  |  |  |  |  |  |  |  |  |  |  |  |  |  |  | 6 |
| 12a |  |  |  |  |  |  |  |  |  |  |  |  |  |  |  |  |  |  |  |  |  |  |  | 6 |
| 12b |  |  |  |  |  |  |  |  |  |  |  |  |  |  |  |  |  |  |  |  |  |  |  | 6 |
|  |  | | | | | | | | | | | | | | | | | | | | | | |  |
| 2a |  |  |  |  |  |  |  |  |  |  |  |  |  |  |  |  |  |  |  |  |  |  |  | 1 |
| 2b |  |  |  |  |  |  |  |  |  |  |  |  |  |  |  |  |  |  |  |  |  |  |  | 1 |
| 4a  ***malT^—^* host** |  |  |  |  |  |  |  |  |  |  |  |  |  |  |  |  |  |  |  |  |  |  |  | 2 |
| 4b |  |  |  |  |  |  |  |  |  |  |  |  |  |  |  |  |  |  |  |  |  |  |  | 1 |
| 5a |  |  |  |  |  |  |  |  |  |  |  |  |  |  |  |  |  |  |  |  |  |  |  | 2 |
| 5b |  |  |  |  |  |  |  |  |  |  |  |  |  |  |  |  |  |  |  |  |  |  |  | 3 |
| 6a |  |  |  |  |  |  |  |  |  |  |  |  |  |  |  |  |  |  |  |  |  |  |  | 1 |
| 6b |  |  |  |  |  |  |  |  |  |  |  |  |  |  |  |  |  |  |  |  |  |  |  | 1 |
| 8a |  |  |  |  |  |  |  |  |  |  |  |  |  |  |  |  |  |  |  |  |  |  |  | 2 |
| 8b |  |  |  |  |  |  |  |  |  |  |  |  |  |  |  |  |  |  |  |  |  |  |  | 2 |

**Supplementary file 1f.** List of mutations in 7-mut OmpF^+^ cI857 lysogen and the two engineered OmpF^—^ genotypes; - λ^-1^ and λ^-2^ (see ‘Coevolutionary replay experiments’ in Methods for details on construction). Bolded mutations represent the three canonical mutations for OmpF^+^ function.

| **λ genotype** | **Mutations** |
| --- | --- |
| **7-mut (OmpF^+^)** | C2999T, **A3034G**, T3230C, C3310T, **G3319A**, **T3321A**, A3364T |
| **λ^-1^ (OmpF^—^)** | C2999T, **A3034G**, T3230C, C3310T, **G3319A**, A3364T |
| **λ^-2^ (OmpF^—^)** | C2999T, T3230C, C3310T, **G3319A**, A3364T |

**Supplementary file 1g.** Pairwise P-value for Tukey’s HSD (honestly significant difference) test comparing difference of means between different competitions in Fig. 3d.

| **Difference of levels** | **Adjusted P-value** |
| --- | --- |
| WT - A | 0.0022 |
| WT - ABC | <0.0001 |
| WT - A**C | <0.0001 |
| A - ABC | <0.0001 |
| A - A**C | <0.0001 |
| ABC - A**C | 0.9998 |

**Supplementary file 1h.** PCR primers for sequencing *J* gene in λ.

| **PCR primers** | |
| --- | --- |
| **Forward Primer (5'-3')** | CCTGCGGGCGGTTTTGTCATTTA |
| **Reverse Primer (3'-5')** | CGCATCGTTCACCTCTCACT |

**Supplementary file 1i.** List of focal mutations with their corresponding two watermark mutations. The three canonical mutations required for OmpF-function (2) are marked in bold.

| Focal *J* Mutation | Mutation ID used for linear regression analysis | Amino Acid Change | Neutral 1 | Neutral 2 |
| --- | --- | --- | --- | --- |
| C2969T | G1 | A → V | G2970C | G2970T |
| A2989G | G2 | I → V | G2985T | C2988T |
| T2991G | G3 | I → M | A2994C | A2994G |
| C2999T | G4 | A → V | G3000A | G3000C |
| A3034G | G5 | S → G | C3033T | T3036A |
| C3310T | G6 | H → Y | G3309A | G3309T |
| G3319A | G7 | D → N | G3315C | G3315A |
| A3320G | G8 | D → G | A3318C | A3318G |
| T3321A | G9 | D → E | T3324A | T3324C |
| T3380C | G10 | L → P | G3378A | G3378C |

**Supplementary file 1j.** Oligos used in MAGE to insert *J* mutations in λ.

| **Mutations**  **introduced** | **Sequence (5'-3')** |
| --- | --- |
| A3321T | CATCGCTGGCAAACGTATACGGCGGAATaTTTGCCGAATACCGTGTGGACGTAAGCGTGAACGTCAGGATCACGTTTCCCCGACCCGCTG |
| G3034A | CATCGGTCACGGTGACAGTACGGGTACCTGACGGCCAGTCCACACtGCTTTCACGCTGGCGCGGAAAAGCCGCGCTCGCCACCTTTACAA |
| 6 ‘wobble’ edits- G3381T C3384A, C3387A, C3390T, G3393A, and C3396A | TAAAACGCCCGTTCCCGGACGAACCTCTGTAACACACTCAtACtACaCTtATtCCaAGCGCCTGTTTCTTAATCACCATAACCTGCACAT |

**Supplementary file 1k.** **PCR primers used to generate *J* amplicons.** These are custom primers designed for the first PCR reaction that uses λ genomic DNA as the template. The second PCR step uses standard primers listed in Kelsic *et al*. (3). Each primer is broken up into three sections. The first, capitalized nucleotides, are the annealing region for the second set of PCR primers. The second, N’s of variable length, improve our ability to multiplex amplicons since they cause reading frame shifts so that when PCRs mixed together originating from different primers there will be variability among the clusters on the Illumina flow cell, allowing the machine to more easily distinguish clusters and reduce sequencing errors. The last segment of lowercase letters indicates the chromosome annealing region. The numbers in the primer label (2949.2968 or 3381.3400) indicate which nucleotides the primer anneals to in *J*. Note, *J* is only 3,399 nucleotides long, so 3400 is one nucleotide beyond its reading frame.

| **Primer label** | **Nucleotide sequence** |
| --- | --- |
| J Mage for 2949.2968 6N illum | CCTACACGACGCTCTTCCGATCTNNNNNNgataaacggtacgctgaggg |
| J Mage for 2949.2968 5N illum | CCTACACGACGCTCTTCCGATCTNNNNNgataaacggtacgctgaggg |
| J Mage for 2949.2968 4N illum | CCTACACGACGCTCTTCCGATCTNNNNgataaacggtacgctgaggg |
| J Mage rev 3381.3400 2N illum | GAGTTCAGACGTGTGCTCTTCCGATCTNNctcagaccacgctgatgccc |
| J Mage rev 3381.3400 1N illum | GAGTTCAGACGTGTGCTCTTCCGATCTNctcagaccacgctgatgccc |
| J Mage rev 3381.3400 0N illum | GAGTTCAGACGTGTGCTCTTCCGATCTctcagaccacgctgatgccc |

**Supplementary file 1l.** *P*-values for two-sample t-tests performed to compare fitness effects of the two neutral markers on the corresponding genotype. Genotypes for sites 5, 6 and 9 had data for only one or none replicate population for one of the neutral markers.

| **Site** | **Single-mutation Genotype** | **P-value** | **Bonferroni corrected P-value** |
| --- | --- | --- | --- |
| G1 | 1000000000 | 0.7676 | 1 |
| G2 | 0100000000 | 0.7940 | 1 |
| G3 | 0010000000 | 0.2535 | 1 |
| G4 | 0001000000 | 0.9053 | 1 |
| G5 | 0000100000 | - | - |
| G6 | 0000010000 | - | - |
| G7 | 0000001000 | 0.2988 | 1 |
| G8 | 0000000100 | 0.7333 | 1 |
| G9 | 0000000010 | - | - |
| G10 | 0000000001 | 0.0088 | 0.0616 |

**References**

1. J. R. Meyer *et al.*, Repeatability and contingency in the evolution of a key innovation in phage lambda. *Science* **335**, 428-432 (2012).

2. R. Maddamsetti *et al.*, Gain-of-function experiments with bacteriophage lambda uncover residues under diversifying selection in nature. *Evolution* **72**, 2234-2243 (2018).

3. E. D. Kelsic *et al.*, RNA Structural Determinants of Optimal Codons Revealed by MAGE-Seq. *Cell Syst* **3**, 563-571 e566 (2016).
